# Patients’ Awareness and Attitudes About the Importance of Sharing Medical History with Dentists in Riyadh, Saudi Arabia

**DOI:** 10.3390/healthcare13212774

**Published:** 2025-10-31

**Authors:** Khalid A. Abalkhail, Sanjeev B. Khanagar, Alanoud Alfawaz, Rand Alharbi, Deem Alsaykhan, Layan Alqahtani

**Affiliations:** 1 Preventive Dental Science Department, College of Dentistry, King Saud Bin Abdulaziz University for Health Sciences, Riyadh 11426, Saudi Arabia; 2 King Abdullah International Medical Research Centre, Ministry of National Guard Health Affairs, Riyadh 11481, Saudi Arabia; 3 College of Dentistry, King Saud bin Abdulaziz University for Health Sciences, Riyadh 11426, Saudi Arabia

**Keywords:** attitude, awareness, disclosure, medical history, patient safety, healthcare, willingness

## Abstract

**Background**: A comprehensive medical history is fundamental to dental care, supporting accurate diagnosis, personalized treatment, and the prevention of adverse outcomes. Despite its importance, patients may underestimate its relevance or hesitate to disclose information to dental professionals. This study aimed to assess patients’ awareness and willingness to disclose medical histories to dental professionals, as well as the effect of an educational intervention on their knowledge. **Methods**: A descriptive cross-sectional study was conducted from 1 December 2023 to 30 September 2024 in Riyadh, Saudi Arabia. Data were collected through a structured bilingual questionnaire assessing demographics, awareness of the importance of medical history, willingness to disclose information, and post-intervention knowledge. The questionnaire’s validity and reliability were established through expert evaluation and test–retest reliability, yielding Aiken’s V values greater than 0.90. Internal consistency was confirmed with a Cronbach’s alpha coefficient of 0.8. A convenience sampling technique was used to recruit the study participants. **Results**: A total of 515 participants completed the survey, with 43.9% withholding medical information from their dentists. Chi-square analysis revealed that disclosure practices were significantly associated with demographic factors, particularly age (*p* ≤ 0.05). Although 90.7% acknowledged the importance of sharing their medical history, only 67.8% reported disclosing it to dental professionals. The regression analysis revealed that participants under 18 years of age (Odds Ratio (OR) 7.08, Confidence Interval (CI) 3.53–50.90) and those aged 18–29 years (OR 14.36, CI 3.45–25.26), as well as participants with elementary (OR 4.55, CI 2.72–20.57) and middle school education levels (OR 4.55, CI 2.72–20.57), were less likely to disclose any underlying medical condition to their dentist. The younger age group (under 18) respondents were significantly more likely to indicate that it was not essential to inform the dentist about their medical condition (OR 6.60, CI 1.05–41.42). Additionally, a significant number of these respondents (OR 5.77, CI 1.87–17.84) reported being unaware of the reasons why dentists request this information, compared to the older age groups. **Conclusions**: The findings reveal a significant knowledge gap concerning patient disclosure of medical history in dental care and highlight the importance of patient education. Implementing targeted educational initiatives is recommended to promote patient disclosure, foster interdisciplinary collaboration, and improve overall patient safety and treatment outcomes.

## 1. Introduction

A medical history provides a comprehensive account of a patient’s health, encompassing both past and present conditions, including illnesses, surgical procedures, medications, allergies, and family health history [1]. This record reflects various lifestyle factors, enabling healthcare professionals to assess potential risks, make precise diagnoses, and tailor treatment plans accordingly. A thorough medical history is essential for effective patient care, as it reveals any health issues that may require intervention [2]. For instance, family medical history can indicate genetic vulnerabilities to ailments such as periodontal disease or oral cancer, thereby aiding in preventive strategies and early detection [3]. Social history includes lifestyle choices such as tobacco and alcohol use, which are significant risk factors for periodontal disease and oral cancer, as well as dietary habits and stress levels that can affect oral health [4]. It is crucial to document allergies, particularly to substances like certain metals, anesthetics, or latex, to prevent severe allergic reactions [1].

Numerous medical conditions manifest with various oral symptoms that can influence the outcomes of dental treatments. Common systemic diseases include diabetes mellitus (DM), hypertension, and autoimmune disorders. In the United States, 7.73% of individuals reported visiting a dentist in the past year without also consulting a medical professional [5]. The American Dental Association recommends that dentists monitor patients’ blood pressure, glucose levels, and cholesterol to assess the risk of heart disease and diabetes mellitus [6]. Additionally, dentists should encourage patients to undergo regular medical check-ups, which facilitate early detection of these conditions and significantly reduce their adverse effects [6]. Patients with poorly controlled diabetes are at increased risk for oral infections, such as periodontitis. Certain infections may extend to fascial spaces, potentially resulting in bacteremia [7]. Furthermore, studies have demonstrated a link between hypertension and periodontitis [8]. Some antihypertensive medications may interact with anesthetics, necessitating their discontinuation prior to general anesthesia [9].

When patients disclose their medications to dentists, it helps in identifying and managing specific oral symptoms. Oral manifestations are also observed in other autoimmune conditions, such as Crohn’s disease and Sjogren’s syndrome. Understanding patients’ medical histories is crucial for establishing exclusion criteria for various diseases [10]. Certain medications can cause oral health issues; therefore, it is essential for patients to inform their dentists about their medication history. For example, bisphosphonates increase the risk of medication-related osteonecrosis of the jaw (MRONJ) and potential implant failures [11]. Additionally, xerostomia is a common oral condition in diabetic patients who are prescribed Metformin. Those on Metformin may also face an increased risk of dental caries, burning mouth syndrome, and delayed wound healing as side effects of the drug [12]. Furthermore, beta blockers, which are commonly used to treat hypertension, can cause burning mouth syndrome and lichenoid reactions [13]. Calcium channel blockers and certain immunosuppressants may lead to gingival hyperplasia [14]. These examples highlight the importance of patients informing their dentists about all medications they are taking to improve treatment outcomes and reduce complications.

There exists a paucity of knowledge regarding the disclosure of medical information to dental practitioners and its implications for diagnosis and treatment, particularly in patients with chronic illnesses [15]. Various factors contribute to patients’ reluctance to share such information, including age differences, perceived stigma, previous experiences with disclosure, trust in dental professionals, and prior interactions with the healthcare system. Currently, there is insufficient data on patients’ awareness of the importance of sharing their medical history with dental professionals. Further research is needed to assess patients’ understanding of medical history relevant to dental care and the prevalence of disclosure among individuals [16]. Obtaining a comprehensive medical history through effective patient communication before commencing dental treatment is essential for providing safe and effective care. Many patients do not recognize the significance of disclosing their medical conditions to dental professionals or the dentist’s role in preventing medical emergencies [17]. Therefore, this study aims to assess patients’ awareness and willingness to share their medical information with their dentists through a structured questionnaire. The objectives were to assess the impact of independent variables like age, gender, educational background on their responses. Additionally, the research also intended to provide the participants with an educational component designed to enhance their awareness regarding the importance of sharing medical history with their dentists.

## 2. Materials and Methods

### 2.1. Research Design

This is a descriptive cross-sectional study conducted from 1 December 2023 to 30 September 2024 in Riyadh, Saudi Arabia. Before initiating the data collection process, ethical clearance was obtained from the Institutional Review Board at the King Abdullah International Medical Research Center in Riyadh, Saudi Arabia (KAIMRC) (IRB approval No. IRB/2995/23, Study No. SP23R/240/11).

### 2.2. Sample Size Estimation

The sample size was estimated based on the data from the pilot survey conducted among 20 patients attending clinics of the College of Dentistry. Based on the responses obtained, the predicted proportion was estimated at 79.6% for the population; the power of the study and confidence interval were 95%, respectively. Based on the above information, the sample size was estimated as 394. The sample size was estimated using G Power software (version 3.1.9.4; G Power, Düsseldorf, Germany).

### 2.3. Sampling Technique

A non-probability convenience sampling method was employed in this study.

### 2.4. Eligibility Criteria

Inclusion criteria: Residents of Saudi Arabia aged 18 years and older who were willing to participate by signing a written informed consent form were included in this study.

Exclusion criteria: Residents who were not citizens or those younger than 18 years of age.

### 2.5. Data Collection Tool

Data were collected using a structured questionnaire consisting of multiple-choice questions organized into five sections. The first section included questions related to demographic data (Q1–Q4), while the second section comprised questions (Q5–Q6) assessing patients’ dental visits and their medical conditions. The third section contained questions evaluating patients’ willingness and awareness regarding the disclosure of their medical history in dental settings (Q7–Q10). The fourth section featured an educational component presented in both Arabic and English, providing information on important aspects of medical history. The final section, the fifth, consisted of questions related to participants’ attitudes and knowledge after reading the educational content. The questionnaire was initially developed in English and later translated into Arabic. A bilingual expert was engaged to carry out the translation, while an independent translator performed a back-translation into English. Collaborative discussions occurred between the translators and the researchers to achieve consensus on the translations. The back-translated version was subsequently compared with the original English questionnaire to ensure the integrity of the questions throughout the translation process.

### 2.6. Validity and Reliability of the Questionnaire

The content validity of the questionnaire was evaluated by a panel of five faculty experts from the College of Dentistry at King Saud bin Abdulaziz University for Health Sciences. All panel members held Master’s or Doctor of Philosophy degrees in Dental Public Health and possessed substantial experience. The primary objective of this assessment was to evaluate the level of agreement on the responses to the questions and to quantify the consistency between panel members for each question using Aiken’s V test. This test is a coefficient of content validity that assesses the relevance, appropriateness, and representativeness of test items. Values greater than 0.90 were achieved for the items included in the questionnaire. For the assessment of reliability, a total of 20 patients attending the College of Dentistry clinics were approached to evaluate the test–retest reliability of the questionnaire. The test–retest reliability was determined by comparing the responses from the same subjects with a four-week interval. Internal consistency was evaluated using Cronbach’s alpha, which yielded a value of 0.8, indicating good reliability.

### 2.7. Data Collection

The data required for this study were collected over a four-month period, from 15 January 2024 to 15 May 2024. Data collection was conducted using a questionnaire that was available in both printed format and as a web-based Google Form. Along with this, an educational leaflet emphasizing the importance of medical history disclosure in dental care was provided to the participants (Supplementary Figure S1). The leaflet was designed to evaluate whether exposure to targeted educational content could modify the attitudes of participants towards disclosure of medical history. This questionnaire was distributed among patients attending dental clinics located in various parts of the city.

### 2.8. Statistical Analysis

The data analysis was conducted using SPSS software, Version 29 (IBM Corporation, Armonk, NY, USA). Descriptive statistics were calculated, and a Chi-square analysis was performed to assess the association between demographic details and participants’ responses. Further regression analysis was conducted to determine the odds ratios associated with each significant finding. Statistical significance was set at *p* ≤ 0.05.

## 3. Results

In the present study, 515 participants responded to the questionnaire. The demographic details of the study participants are presented in Table 1. Most participants, 410 (79.6%), were from the Al-Riyadh region, while lesser percentages were from other regions. Most of the participants, 202 (39.2%), were within the age group of 18–29 years, followed by those aged 45–60 years, 130 (25.2%). A total of 307 (59.6%) of the participants were female, and 208 (40.4%) were male. A significant percentage of participants were college graduates, 312 (60.6%), followed by high school graduates, 115 (22.3%), postgraduate studies, 53 (10.3%), middle school, 21 (4.10%) and elementary school, 14 (2.70%).

Details of participants’ responses are presented in Table 2. The data indicate that most study participants, 394 (76.5%), visited a dentist in the previous year, with a substantial proportion, 342 (66.4%), having been inquired about their medical history. Surprisingly, 289 (43.9%) of the participants chose not to disclose their medical conditions to the dentists during their appointments. However, 467 (90.7%) of the participants considered it essential to notify dentists about medical issues, and 349 (67.8%) understood the necessity of doing so. However, 166 (32.20%) participants did not know the reason why dentists inquire about their medical conditions.

Details of the participants’ responses after viewing the educational information are presented in Table 3. After viewing the educational material, 493 (95.7%) of the study participants acknowledged the importance of disclosing their medical conditions to dentists, and 506 (98.3%) expressed their willingness to provide this information to their dentists in the future.

The association between participants’ place of residence and their responses is presented in Table 4. Although most individuals expressed favorable views and behaviors regarding sharing their medical information with dentists, some variability in practices and awareness exists across regions. The statistically significant correlation between the region and reported health problems highlights potential regional health disparities that warrant further investigation. The calculated *p*-value of 0.03, which is below the established significance level of 0.05, resulted in the rejection of the null hypothesis. This indicates that the prevalence of medical issues reported by participants differed significantly by geographic location. Specifically, the prevalence of diabetes and hypertension varied by region, demonstrating a significant correlation between place of residence and reported medical problems (*p* = 0.03). Diabetes prevalence was highest in the Southern region at 10 (23.8%), whereas the “no medical condition” category was most prevalent in the Al-Qassim and Western regions, with 13 (81.2%) and 22 (81.5%), respectively.

The association between participants’ age and their responses is presented in Table 5. The data indicate significant correlations between age and behaviors related to disclosing medical history to dental professionals. Younger participants, particularly those under 18 and those aged 18–29, were less likely to reveal medical concerns and demonstrated lower awareness about the reasons dentists inquire about such conditions. The responses of the younger age groups to the second question predominantly indicated the absence of medical issues, with frequencies of 18 (85.7%) and 186 (92.1%), respectively. Although almost all participants recognized the importance of sharing their medical history, the younger population showed slightly less consensus. Only 9 (42.9%) of those under 18 and 90 (44.6%) of those aged 18–29 reported disclosing their medical conditions to their dentists. On the contrary, older individuals were more likely to have medical illnesses, disclose these conditions during their dental appointments, and understand the importance of providing such information. Participants aged over 60 exhibited the highest level of disclosure, with 43 (89.6%) reporting their medical issues during dental appointments. The prevalence of diabetes and hypertension increased with older age, peaking among individuals over 60, at 12 (25.0%) and 13 (27.1%), respectively, reflecting the growing burden of chronic illnesses in older populations. Older participants were also more likely to consider sharing medical history important, with 46 (95.8%) of those over 60 agreeing, compared to 16 (76.2%) of participants under 18. The understanding of why dentists request medical information increased with age, rising from 9 (42.9%) among participants under 18 to 39 (81.2%) among those over 60. These findings highlight the need to increase awareness among younger patients about the significance of medical history in dental treatment.

The association between participants’ gender and their responses is presented in Table 6. The findings indicate that while males reported higher incidences of certain medical conditions (e.g., diabetes and heart disease), both genders exhibited similar attitudes and behaviors regarding disclosure of medical history and its importance in dental care. Most responses showed no statistically significant differences, suggesting generally comparable attitudes and practices between genders. The only significant difference was observed in the reported medical problems. The prevalence of diabetes was higher in males at 28 (13.5%) compared to 15 (4.9%) in females. Males had a heart disease incidence of 7 (3.4%), while females had a higher incidence of asthma at 12 (3.9%) compared to 3 (1.4%) in males. Additionally, females reported a higher overall rate of absence of any medical issues, with 235 (76.5%) compared to 138 (66.3%) in males.

The association between participants’ educational level and their responses is presented in Table 7. A significant correlation was observed between educational levels and reported medical conditions. The prevalence of diabetes and hypertension was higher among individuals with lower educational levels: 2 (14.3%) and 4 (28.6%), respectively, among those with only an elementary school education, compared to 17 (5.4%) and 23 (7.4%) among college graduates. Interestingly, postgraduate participants reported elevated incidences of both diabetes and hypertension, each at 10 (18.9%). Disclosure of medical conditions during dental visits was also more common among participants with lower education levels; for example, 16 (76.2%) among those with middle school degrees disclosed their conditions, compared to 163 (52.2%) of college graduates. Despite these differences, most participants across all educational levels acknowledged the significance of disclosing their medical history to their dentist and expressed a willingness to do so in the future. Understanding of the rationale behind dentists inquiring about medical history improved with higher education levels, indicating an opportunity for focused awareness efforts among less-educated populations.

The multivariate logistic regression analysis presented in Table 8 revealed that all the age groups—less than 18 years (OR 7.08, CI 2.05–24.38), 18–29 years (OR 14.36, CI 6.86–30.06), 30–44 years (OR 4.55, CI 2.22–9.34), and 45–60 years (OR 2.13, CI 1.82–4.21)—were less likely to have any underlying medical condition compared to those over 60 years of age. When gender was analyzed, females (OR 1.79, CI 1.21–2.65) were less likely to have any underlying medical condition. Assessment by education level showed that individuals with a high school (OR 2.83, CI 1.42–5.61) or college-level education (OR 3.64, CI 1.98–6.68) were less likely to have any medical condition than postgraduate respondents.

In response to the question “During your last visits to the dentist, did you mention your medical condition?”, it was revealed that the younger age groups—under 18 years (OR 7.08, CI 3.53–50.90) and 18–29 years (OR 14.36, CI 3.45–25.26)—were less likely to disclose any underlying medical condition compared to adults aged 30–44 (OR 4.55, CI 2.72–20.57) and 45–60 (OR 2.13, CI 1.39–10.43). When the same question was analyzed for significance based on educational level, it was revealed that participants with elementary (OR 4.55, CI 2.72–20.57) and middle school education (OR 4.55, CI 2.72–20.57) were less likely to mention their underlying medical condition to the dentist.

The younger age group (under 18) respondents were significantly more likely to indicate that it was not essential to inform the dentist about their medical condition (OR 6.60, CI 1.05–41.42). Additionally, a significant number of these respondents (OR 5.77, CI 1.87–17.84) reported being unaware of the reasons why dentists request this information, compared to the older age groups.

## 4. Discussion

Despite the high prevalence of self-reported medical conditions among individuals seeking dental care, evidence suggests that some patients do not disclose their medical histories to their dentists [16]. A significant number of patients seeking dental care do not fully disclose their medical histories, posing potential health risks. Factors contributing to this include a lack of awareness about the link between dental care and medical issues, fear of being judged, limited health literacy, and language barriers, which are potential reasons for this underreporting [16]. In the present study, 33% of participants reported that their dentist did not inquire about their medical history, highlighting the responsibility of dental professionals to actively obtain this information. To address this issue, dental practitioners should implement comprehensive patient intake forms, conduct thorough follow-up inquiries, and create a setting that promotes open communication to address this problem. More efficient information exchange can be achieved through collaboration with physicians and integrating electronic health records. To ensure safe and efficient dental care, patient education regarding the significance of sharing medical history must be improved.

The details of the responses of the current study indicated that the majority of study participants (76.5%) had visited a dentist in the previous year. These findings were contrary to another study by Alonaizan et al., which revealed that only 21.8% of participants reported visiting a dental hospital during the same period [18]. This difference could be attributed to various factors, including patients’ education levels, disparities in access to care, and cost. In the current study, 70.9% of participants had attained college or higher degrees. However, there was no positive correlation between advanced education level and regular attendance of dental visits. On the other hand, a recent study by Almutairi et al. showed a higher likelihood of regular visits to dental practitioners among individuals with a college education or above, comprising 29.2% of respondents [19]. This discrepancy may be caused by variations in the study samples. Dental care utilization rates may be influenced by demographic factors, including age, socioeconomic status, education level, and geographic location. For example, compared to research done in rural or underprivileged populations, studies involving individuals from urban areas with better access to private clinics might reveal higher dental attendance rates. Similarly, while lower-income groups may depend more on public or hospital-based treatments, higher-income populations may have more financial freedom to seek private care [15].

In this study, findings indicate that 27.6% of participants suffer from medical conditions, similar to those in the Alonaizan et al. study done in Eastern Saudi Arabia, which showed a similar percentage of 25.7% [18]. Conversely, a study from the Southwest region of Saudi Arabia reported a higher prevalence of medical conditions (40.2%) among patients seeking periodontal treatment [20]. In contrast, a review of patient data from periodontal clinics at King Saud University, Riyadh, Saudi Arabia, by Almas and Awartani (2003) [21] found that only 10% of the patient population had systemic disorders. The higher prevalence of medical disorders observed in the current research may be attributed to the increased prevalence of diabetes mellitus and its comorbidities over the past few decades, as compared to estimates by Almas et al. in 2003 [21].

In the current study, diabetes and hypertension were the most prevalent medical conditions, affecting 8.3% and 8.5% of participants, respectively. These findings are consistent with those of similar studies conducted in Saudi Arabia [22,23]. The prevalence of diabetes in Saudi Arabia has been increasing over the years, reaching a rate of 18.7% in 2021, according to the data from the International Diabetes Federation [24]. This rise in diabetes prevalence is largely attributed to the increased obesity rates and other predisposing factors. Among diabetic patients, hypertension and dyslipidemia are most the common risk factors for cardiovascular diseases, and 70–75% of patients with coronary artery disease also have diabetes or abnormal glucose levels [25,26].

Remarkably, 56.1% of individuals chose to disclose their medical issues to dentists, compared to 61.3% reported in a study conducted by Bin Mubayrik et al. [27]. This discrepancy could be attributed to various factors, including the demographic characteristics of the study participants. In the current study, 90.7% of participants stated that they believed it was crucial to disclose their health concerns to dentists, indicating a high level of awareness among the sample. However, this contrasts with their actual willingness to disclose their medical information. Patients’ unwillingness to share their medical history might be caused by privacy concerns, fear of stigma, or unpleasant past experiences with healthcare providers. These barriers can be addressed not only by educating patients about the importance of sharing their medical information but also by explaining the reasons behind this necessity. This can enhance the level of basic health literacy by providing access to reliable and accurate information [28]. Strengthening the patient–dentist relationship, promoting effective communication, and ensuring patient privacy are essential for creating a safe environment for the patients. In the current research, we analyzed the existing behavior or awareness in relation to post-reading intentions. The majority of participants indicated their readiness to share this information with their dentists in the future. Nevertheless, this should not be regarded as a causal influence, as the paper does not quantify within-subject changes, and these are not substantiated by statistical analysis. These stated limitations, along with convenience sampling, can restrict the generalizability of our study results.

There were some limitations in our study. The use of self-reported data for study variables could have introduced bias in our findings. This includes social desirability bias, where participants would respond in a manner they believe is socially acceptable rather than being truthful. Recall bias is a potential limitation in this study, where respondents are subject to forgetting certain information. Additionally, misinterpretation of the questions can affect the accuracy of the responses when questions lack clarity. Moreover, compared to the sample that was excluded because of non-response, the overrepresentation of educated participants may not reflect the general population and might reflect selection bias. Although a portion of both genders and all educational classes were still included in the sample, this might have added non-response bias. Furthermore, despite its effectiveness, digital distribution may leave out those without access to technology or who are less familiar with using digital tools, which could lead to the underrepresentation of some demographic groups, particularly the elderly and those from lower socioeconomic backgrounds. Although this study provides valuable information on patient perceptions of medical history disclosure in dental care, addressing these limitations in future research would increase understanding of this critical issue and improve communication between patients and dental care providers.

## 5. Conclusions

In the present study, most participants acknowledged the importance of sharing their medical history; however, a significant proportion did not disclose information regarding their medical conditions to their dentists. Younger participants, as well as those with elementary and middle school education levels, were less likely to disclose any underlying medical conditions to their dentist. Additionally, many participants were unaware of the reasons why dentists inquire about their medical conditions. These findings highlight the necessity for multifaceted strategies that extend beyond raising awareness alone, incorporating approaches that actively motivate patients to engage in full and transparent communication with their dental providers. Strengthening interprofessional collaboration between dental and medical practitioners can enhance patient education and reinforce the link between systemic health and oral care. In addition, structured educational initiatives, culturally tailored interventions, and improved dentist–patient communication may help reduce disparities in disclosure practices. Such measures are critical not only for ensuring patient safety and accurate treatment planning but also for advancing overall health outcomes through more effective integration of medical and dental care.

## Figures and Tables

**Table 1:** Distribution of study participants based on demographic details.

Statements	Responses	Frequency	Percentage
**Place of residence**	Al-Qassim region	16	3.10
	Al-Riyadh region	410	79.60
	Eastern region	14	2.70
	Northern region	6	1.20
	Southern region	42	8.20
	Western region	27	5.20
**Age**	Under 18 years	21	4.10
	18–29 years	202	39.20
	30–44 years	114	22.10
	45–60 years	130	25.20
	More than 60 years	48	9.30
**Gender**	Female	307	59.60
	Male	208	40.40
**Educational level**	College graduate	312	60.60
	Elementary school	14	2.70
	High School	115	22.30
	Middle School	21	4.10
	Postgraduate studies	53	10.30

**Table 2:** Details of the study participants’ responses to the questions.

Sl. No.	Statements	Responses	Frequency	Percentage
Q1	Have you visited the dentist in the last 12 months?	No	121	23.50
		Yes	394	76.50
Q2	Do you have any medical conditions?	Diabetes	43	8.30
		Hypertension	44	8.50
		No medical condition	373	72.40
		Heart disease	9	1.70
		Asthma	15	2.90
		Others	31	6.00
Q3	Have you ever been asked about your medical history by your dentist?	No	173	33.60
		Yes	342	66.40
Q4	During your last visits to the dentist, did you mention your medical condition?	No	226	43.90
		Yes	289	56.10
Q5	Do you consider it important to tell the dentist about your medical condition?	No	48	9.30
		Yes	467	90.70
Q6	Do you know why the dentist asks you about this information?	No	166	32.20
		Yes	349	67.80

**Table 3:** Details of the participants’ responses after viewing the educational information.

Sl. No.	Statements	Responses	Frequency	Percentage
1	Do you now think it is important to share your medical history with the dentist?	No	22	4.30
		Yes	493	95.70
2	In the future, are you willing to share your medical information with your dentist?	No	9	1.70
		Yes	506	98.30

**Table 4:** Association of place of residence of the participants with their responses.

Statements and Responses		Place of Residence												χ^2^ Value	*p* Value
		Al-Qassim Region		Al-Riyadh Region		Eastern Region		Northern Region		Southern Region		Western Region			
		N	%	N	%	N	%	N	%	N	%	N	%		
Q1	No	5	31.20	93	22.70	1	7.10	1	16.70	13	31.00	8	29.60	4.78	0.442
	Yes	11	68.80	317	77.30	13	92.90	5	83.30	29	69.00	19	70.40		
Q2	Diabetes	2	12.50	30	7.30	0	0.00	0	0.00	10	23.80	1	3.70	39.84	0.03 *
	Hypertension	1	6.20	37	9.00	0	0.00	1	16.70	4	9.50	1	3.70		
	No medical condition	13	81.20	297	72.40	10	71.40	5	83.30	26	61.90	22	81.50		
	Heart disease	0	0.00	6	1.50	2	14.30	0	0.00	1	2.40	0	0.00		
	Asthma	0	0.00	13	3.20	0	0.00	0	0.00	1	2.40	1	3.70		
	Others	0	0.00	27	6.60	2	14.30	0	0.00	0	0.00	2	7.40		
Q3	No	8	50.00	133	32.40	1	7.10	2	33.30	19	45.20	10	37.00	9.26	0.099
	Yes	8	50.00	277	67.60	13	92.90	4	66.70	23	54.80	17	63.00		
Q4	No	11	68.80	178	43.40	5	35.70	2	33.30	16	38.10	14	51.90	5.97	0.309
	Yes	5	31.20	232	56.60	9	64.30	4	66.70	26	61.90	13	48.10		
Q5	No	1	6.20	41	10.00	1	7.10	0	0.00	3	7.10	2	7.40	1.45	0.919
	Yes	15	93.80	369	90.00	13	92.90	6	100.00	39	92.90	25	92.60		
Q6	No	6	37.50	133	32.40	1	7.10	3	50.00	14	33.30	9	33.30	5.15	0.398
	Yes	10	62.50	277	67.60	13	92.90	3	50.00	28	66.70	18	66.70		

* Statistical significance set at 0.05; N: Number of samples; χ^2^ Value: Chi square value.

**Table 5:** Association of age of the participants with their responses.

Statements and Responses		Age										χ^2^ Value	*p* Value
		Under 18		18–29		30–44		45–60		More Than 60			
		N	%	N	%	N	%	N	%	N	%		
Q1	No	4	19.00	50	24.80	31	27.20	29	22.30	7	14.60	3.49	0.478
	Yes	17	81.00	152	75.20	83	72.80	101	77.70	41	85.40		
Q2	Diabetes	1	4.80	4	2.00	10	8.80	16	12.30	12	25.00	129.71	0.001 *
	Hypertension	0	0.00	1	0.50	9	7.90	21	16.20	13	27.10		
	No medical condition	18	85.70	186	92.10	83	72.80	69	53.10	17	35.40		
	Heart disease	0	0.00	0	0.00	0	0.00	6	4.60	3	6.20		
	Asthma	0	0.00	4	2.00	3	2.60	8	6.20	0	0.00		
	Others	2	9.50	7	3.50	9	7.90	10	7.70	3	6.20		
Q3	No	5	23.80	75	37.10	42	36.80	38	29.20	13	27.10	4.59	0.332
	Yes	16	76.20	127	62.90	72	63.20	92	70.80	35	72.90		
Q4	No	12	57.10	112	55.40	56	49.10	41	31.50	5	10.40	43.61	0.001 *
	Yes	9	42.90	90	44.60	58	50.90	89	68.50	43	89.60		
Q5	No	5	23.80	25	12.40	8	7.00	8	6.20	2	4.20	11.21	0.024 *
	Yes	16	76.20	177	87.60	106	93.00	122	93.80	46	95.80		
Q6	No	12	57.10	66	32.70	40	35.10	39	30.00	9	18.80	10.7	0.03 *
	Yes	9	42.90	136	67.30	74	64.90	91	70.00	39	81.20		
	No	21	100.00	196	97.00	111	97.40	130	100.00	48	100.00		

* Statistical significance set at 0.05; N: Number of samples; χ^2^ Value: Chi square value.

**Table 6:** Association of gender of the participants with their responses.

Statements and Responses		Gender				χ^2^ Value	*p* Value
		Female		Male			
		N	%	N	%		
Q1	No	71	23.10	50	24.00	0.05	0.811
	Yes	236	76.90	158	76.00		
Q2	Diabetes	15	4.90	28	13.50	21.02	0.001 *
	Hypertension	24	7.80	20	9.60		
	No medical condition	235	76.50	138	66.30		
	Heart disease	2	0.70	7	3.40		
	Asthma	12	3.90	3	1.40		
	Others	19	6.20	12	5.80		
Q3	No	109	35.50	64	30.80	1.24	0.264
	Yes	198	64.50	144	69.20		
Q4	No	139	45.30	87	41.80	0.59	0.439
	Yes	168	54.70	121	58.20		
Q5	No	23	7.50	25	12.00	3.00	0.083
	Yes	284	92.50	183	88.00		
Q6	No	96	31.30	70	33.70	0.32	0.57
	Yes	211	68.70	138	66.30		
	Yes	302	98.40	204	98.10		

* Statistical significance set at 0.05; N: Number of samples; χ^2^ Value: Chi square value.

**Table 7:** Association of educational level of the participants with their responses.

Statements and Responses		Educational Level										χ^2^ Value	*p* Value
		College Graduate		Elementary School		High School		Middle School		Postgraduate Studies			
		N	%	N	%	N	%	N	%	N	%		
Q1	No	72	23.10	2	14.30	35	30.40	3	14.30	9	17.00	6.01	0.198
	Yes	240	76.90	12	85.70	80	69.60	18	85.70	44	83.00		
Q2	Diabetes	17	5.40	2	14.30	8	7.00	6	28.60	10	18.90	64.91	0.001 *
	Hypertension	23	7.40	4	28.60	6	5.20	1	4.80	10	18.90		
	No medical condition	245	78.50	7	50.00	86	74.80	8	38.10	27	50.90		
	Heart disease	2	0.60	0	0.00	5	4.30	1	4.80	1	1.90		
	Asthma	11	3.50	0	0.00	1	0.90	1	4.80	2	3.80		
	Others	14	4.50	1	7.10	9	7.80	4	19.00	3	5.70		
Q3	No	107	34.30	4	28.60	37	32.20	5	23.80	20	37.70	1.64	0.802
	Yes	205	65.70	10	71.40	78	67.80	16	76.20	33	62.30		
Q4	No	149	47.80	4	28.60	52	45.20	5	23.80	16	30.20	10.78	0.029 *
	Yes	163	52.20	10	71.40	63	54.80	16	76.20	37	69.80		
Q5	No	28	9.00	3	21.40	10	8.70	3	14.30	4	7.50	3.33	0.503
	Yes	284	91.00	11	78.60	105	91.30	18	85.70	49	92.50		
Q6	No	104	33.30	6	42.90	37	32.20	8	38.10	11	20.80	4.42	0.352
	Yes	208	66.70	8	57.10	78	67.80	13	61.90	42	79.20		

* Statistical significance set at 0.05; N: Number of samples; χ^2^ Value: Chi square value.

**Table 8:** Multivariate logistic regression analysis of the participants’ demographic details with their responses.

Variable	Independent Variable	B	df	*p*-Value	Exp(B)	95% Confidence Interval for Exp(B)	
						Lower Bound	Upper Bound
**Do you have any medical condition?**	Under 18	1.95	1	<0.0001 *	7.08	2.05	24.38
	18–29	2.66	1	<0.0001 *	14.36	6.86	30.06
	30–44	1.51	1	<0.0001 *	4.55	2.22	9.34
	45–60	0.75	1	0.02 *	2.13	1.82	4.21
	More than 60	0 ^#^	-	-	-	-	-
	Female	0.58	1	<0.0001 *	1.79	1.21	2.65
	Male	0 ^#^	-	-	-	-	-
	College graduate	1.29	1	<0.0001 *	3.64	1.98	6.68
	Elementary school	0.00	1	1.00	1.00	0.30	3.25
	High school	1.04	1	<0.0001 *	2.83	1.42	5.61
	Middle school	−0.28	1	0.58	0.75	0.27	2.08
	Postgraduate studies	0 ^#^	-	-	-	-	-
**During your last visits to the dentist did you mention your medical condition?**	Under 18	2.59	1	<0.0001 *	7.08	3.53	50.90
	18–29	2.23	1	<0.0001 *	14.36	3.45	25.26
	30–44	2.01	1	<0.0001 *	4.55	2.72	20.57
	45–60	1.33	1	<0.0001 *	2.13	1.39	10.43
	More than 60	0 ^#^	-	-	-	-	-
	College graduate	0.19	1	0.57	1.22	0.61	2.42
	Elementary school	−0.37	1	0.61	0.69	0.16	2.92
	High school	0.07	1	0.84	1.07	0.50	2.27
	Middle school	−0.51	1	0.43	0.60	0.16	2.13
	Postgraduate studies	0 ^#^	-	-	-	-	-
**Do you consider it important to tell the dentist about your medical condition?**	Under 18	1.88	1	0.04 *	6.60	1.05	41.42
	18–29	1.50	1	0.06	4.48	0.93	21.59
	30–44	0.81	1	0.33	2.25	0.43	11.61
	45–60	0.52	1	0.52	1.68	0.33	8.40
	More than 60	0 ^#^	-	-	-	-	-
**Do you know why the dentist asks you about this information?**	Under 18	0.37	1	<0.0001 *	5.77	1.87	17.84
	18–29	1.75	1	0.06	2.10	0.96	4.59
	30–44	0.39	1	0.04 *	2.34	1.03	5.32
	45–60	0.41	1	0.13	1.85	0.82	4.20
	More than 60	0 ^#^	-	-		-	-

^#^—Reference category; last category was considered the reference; df—degree of freedom, B—odds; and * significance = (*p* < 0.05).

## Data Availability

All the data of this research are included and presented in the article.

## Supplementary Materials

The following supporting information can be downloaded at: https://www.mdpi.com/article/10.3390/healthcare13212774/s1, Figure S1: Educational Leaflet.
