# Supplementary material for: Patients’ Awareness and Attitudes About the Importance of Sharing Medical History with Dentists in Riyadh, Saudi Arabia
Source: Healthcare (Basel). 2025 Oct 31;13(21):2774. doi: 10.3390/healthcare13212774 (PMC12607543; doi:10.3390/healthcare13212774)
Supplement: Supplementary file 1 [file healthcare-13-02774-s001.zip › healthcare-3917674-supplementary.pdf]

## ما هي المعلومات التي يجب على المريض مشاركتها مع طبيب الاسنان؟

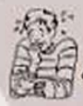

حالة المريض وصحته النفسية

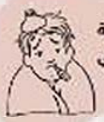

حالة المريض الصحية او امراض مزمنه يعاني منها

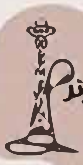

عادات يومية تؤثر على الصحة

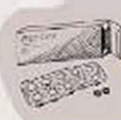

نوع وجرعة أي دواء إن وجد

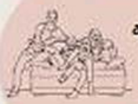

الامراض العائلية والوراثية

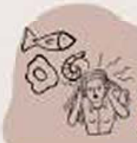

حساسية من طعام او دواء

## لماذا يجب علينا مشاركة طبيب الاسنان بهذه المعلومات؟!

طبيب الاسنان يستطيع معرفة الكثير من الكشف المبدئي لكن دور المريض الفعّال ومساعدة بمشاركة هذه المعلومات ستضيف لطبيب الاسنان الكثير بحيث سيكون من الاسهل تجنب اي مضاعفات بسبب تعارض بعض الادوية او سوء استخدام المواد التي من الممكن ان تضر المريض من الممكن كذلك تفادي ردات فعل محتمله و التقليل من فرص فشل العلاج مما سيحسن من فرص نجاح العلاج والتوصل لنتيجة مرضية لكلا الطرفين وتاكد عزيزي المراجع ان الطبيب سيحفظ لك خصوصيتك.

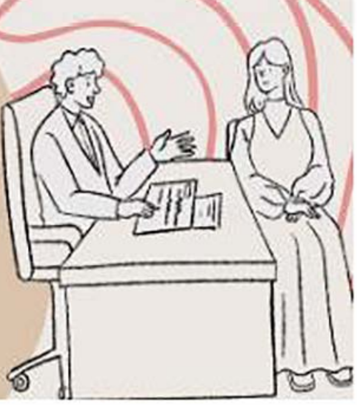

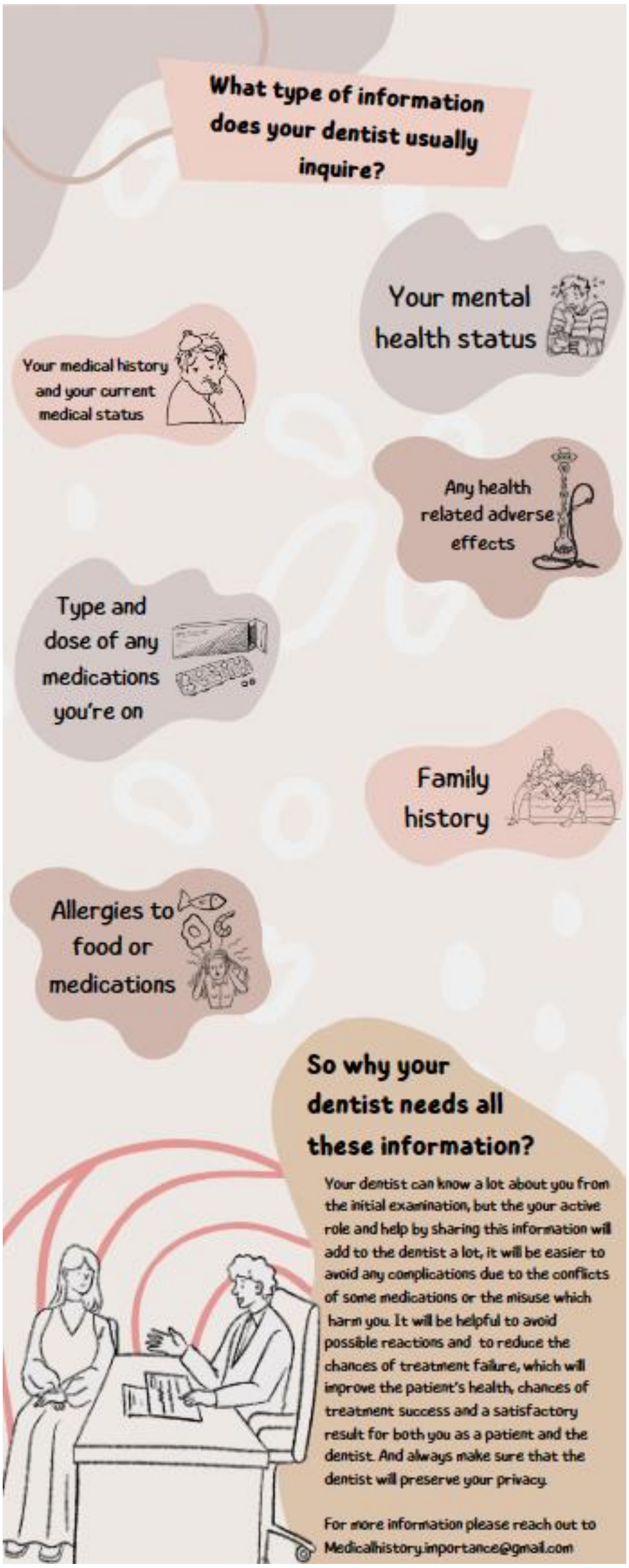


Figure S1: Educational Leaflet.
